# Network Pharmacology-Driven Sustainability: AI and Multi-Omics Synergy for Drug Discovery in Traditional Chinese Medicine

**DOI:** 10.3390/ph18071074

**Published:** 2025-07-21

**Authors:** Lifang Yang, Hanye Wang, Zhiyao Zhu, Ye Yang, Yin Xiong, Xiuming Cui, Yuan Liu

**Affiliations:** 1 Center for Translational Research in Clinical Medicine, School of Medicine, Kunming University of Science and Technology, Kunming 650500, China; ylfkmust1990@163.com; 2 Faculty of Life Science and Technology, Kunming University of Science and Technology, Kunming 650500, China; wanghanyekust@163.com (H.W.); zyao1205@163.com (Z.Z.); yangye@kust.edu.cn (Y.Y.); yhsiung@163.com (Y.X.); 3 Department of Pharmacy, Xi’an Aerospace Hospital, Xi’an 710100, China; 4 Key Laboratory of *Panax notoginseng* Resources Sustainable Development and Utilization of State Administration of Traditional Chinese Medicine, Kunming 650500, China; 5 Yunnan Provincial Key Laboratory of *Panax notoginseng*, Kunming 650500, China; 6 Kunming Key Laboratory of Sustainable Development and Utilization of Famous-Region Drug, Kunming 650500, China; 7 Sanqi Research Institute of Yunnan Province, Kunming 650500, China

**Keywords:** traditional Chinese medicine, network pharmacology, artificial intelligence, multi-omics, sustainable drug discovery

## Abstract

Traditional Chinese medicine (TCM), a holistic medical system rooted in dialectical theories and natural product-based therapies, has served as a cornerstone of healthcare systems for millennia. While its empirical efficacy is widely recognized, the polypharmacological mechanisms stemming from its multi-component nature remain poorly characterized. The conventional trial-and-error approaches for bioactive compound screening from herbs raise sustainability concerns, including excessive resource consumption and suboptimal temporal efficiency. The integration of artificial intelligence (AI) and multi-omics technologies with network pharmacology (NP) has emerged as a transformative methodology aligned with TCM’s inherent “multi-component, multi-target, multi-pathway” therapeutic characteristics. This convergent review provides a computational framework to decode complex bioactive compound–target–pathway networks through two synergistic strategies, (i) NP-driven dynamics interaction network modeling and (ii) AI-enhanced multi-omics data mining, thereby accelerating drug discovery and reducing experimental costs. Our analysis of 7288 publications systematically maps NP-AI–omics integration workflows for natural product screening. The proposed framework enables sustainable drug discovery through data-driven compound prioritization, systematic repurposing of herbal formulations via mechanism-based validation, and the development of evidence-based novel TCM prescriptions. This paradigm bridges empirical TCM knowledge with mechanism-driven precision medicine, offering a theoretical basis for reconciling traditional medicine with modern pharmaceutical innovation.

## 1. Introduction

Traditional Chinese medicine (TCM), a comprehensive medical system refined through millennia of clinical practice, serves as the primary healthcare modality for one-quarter of the global population [1,2]. Despite the botanical origins of life-saving drugs including artemisinin, paclitaxel, and digoxin, the phytochemical diversity and structural complexity inherent in herbal matrices have impeded the mechanistic elucidation of their pharmacological actions and hindered global acceptance [3]. Three systemic challenges currently constrain research on bioactive ingredients in TCM prescriptions. First, phytochemical characterization faces analytical limitations—single herbs like *Salvia miltiorrhiza* harbor over 100 structurally analogous diterpenoids that challenge conventional GC-MS and LC-MS/MS differentiation [4]. Second, the “Jun (Monarch)–Chen (Minister)–Zuo (Assistant)–Shi (Guide)” formulation philosophy achieves therapeutic holism through dynamic multi-target modulation but obscures causal relationships between specific components and clinical outcomes [5]. Third, conventional trial-and-error approaches incur unsustainable costs, including time-intensive bioassays and excessive solvent consumption during the screening of the bioactive compounds in TCM herbs and prescriptions [6].

Network pharmacology (NP) has emerged as a pivotal paradigm to address these challenges. By constructing multidimensional “herb–component–target–disease” networks, NP aligns with TCM’s holistic philosophy to systematically decode multi-component, multi-target, and multi-pathway mechanisms [7]. Key strategies include database-driven screening (e.g., TCMSP), molecular docking validation of target interactions (e.g., inflammatory factors), and pathway enrichment analysis (e.g., PI3K/AKT) to prioritize bioactive candidates such as flavonoids and saponins [8,9,10]. Artificial intelligence (AI) further revolutionizes NP by enabling predictive precision through two approaches: graph neural networks (GNNs) analyze complex component–target–disease networks, while AlphaFold3 predicts protein structures to optimize molecular docking [11,12]. The AI-driven platform Chemistry42 exemplifies how generative AI facilitates molecular design and optimization, enabling the structural refinement of novel derivatives for enhanced therapeutic efficacy and attenuated toxicity [13]. Collective, these tools minimize reliance on trial-and-error approaches, significantly reduce resource consumption in screening workflows, and accelerate drug discovery for complex and chronic diseases. The advent of multi-omics technologies (e.g., transcriptomics, proteomics, metabolomics) further empowers NP with multidimensional validation, enabling systematic drug discovery from TCM prescriptions [14]. Transcriptomics reveals gene co-expression networks, proteomics maps disease-related protein networks influenced by bioactive components, and metabolomics rapidly identifies active molecules, while multi-omics integration with NP constructs dynamic “component–target–phenotype” networks [15,16,17]. For instance, by integrating NP with transcriptomic, proteomic, and metabolomic profiling, Li et al. demonstrated that the Jianpi-Yishen formula attenuates chronic kidney disease progression through betaine-mediated regulation of glycine/serine/threonine metabolism coupled with tryptophan metabolic reprogramming, synergistically modulating M1/M2 macrophage polarization dynamics to restore inflammatory microenvironment homeostasis [18].

The convergence of NP, AI, and multi-omics now represents the optimal paradigm for screening bioactive compounds in TCM prescriptions. NP provides a systemic framework, AI accelerates target prediction and molecular optimization, and multi-omics offers high-throughput mechanistic validation. This triad synergistically deciphers TCM’s “black box” through computational prioritization and experimental refinement, bridging empirical knowledge with mechanism-driven precision. By harmonizing these technologies, researchers can sustainably unlock TCM’s therapeutic potential while advancing its global scientific legitimacy.

Through the analysis of 7288 publications, this review systematically synthesizes (i) computational resources (databases and analytical pipelines) for NP analysis, (ii) convergent applications via AI-powered prediction of phytochemical–disease target interactions validated by multi-omics profiling, and (iii) challenges and prospects in scaling NP-AI–omics integration for herbal drug discovery. Furthermore, we propose an integrated NP-AI–omics workflow for the continuous discovery of novel bioactive compounds in TCM prescriptions. These insights will empower researchers to apply integrated methodologies for screening pharmacologically active novel monomers and developing innovative TCM prescriptions.

## 2. Data Collection and Analysis Processing

**Figure 1:**
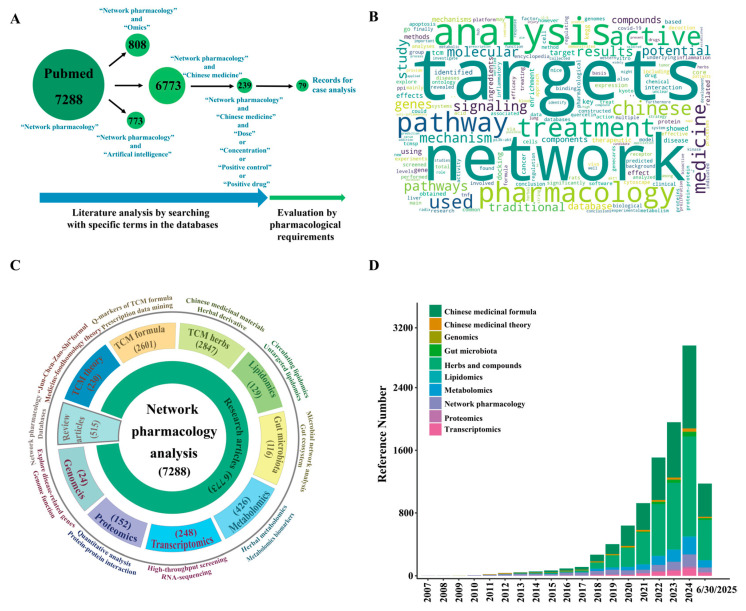
Summary of literature analysis on traditional Chinese medicine. (**A**) Analysis pipeline of the network analysis/pharmacology/medicine literature related to traditional Chinese medicine published from 2007 to the first half of 2025 in PubMed. (**B**) Word cloud analysis results of abstracts from 7288 publications. (**C**) Research method classification of network analysis/pharmacology/medicine in traditional Chinese medicine: TCM theory mainly involves TCM traditional compatibility theory and medicine–food homology theory. TCM formula studies include quality markers (Q-markers) of TCM formulas, prescription data mining, and pharmacology of classic prescriptions. TCM herbs focus on the pharmacology of herbal derivatives and Chinese medicinal material. Network pharmacology combined with omics can comprehensively analyze the metabolism, absorption, and action mechanisms of Chinese medicine, involving circulating lipidomics, protein–protein interaction, gut microbial network analysis, untargeted metabolomics, high-throughput screening, genome functional analysis, and exploration of disease key genes. (**D**) Network pharmacology research articles on traditional Chinese medicine published from 2007 to the first half of 2025 in the PubMed database.

The PubMed database was searched using the term “network pharmacology” in the text word field (1 January 2007 to 30 June 2025), excluding non-English records, and records available in full text were included. The terms “network pharmacology”, “artificial intelligence”, and “omics” were used to search the PubMed database for data related to the combination of two or three approaches (Figure 1A). First, the combined terms “network pharmacology” and “Chinese medicine” were used to gain a general understanding of this field. Second, the term combination “network pharmacology” and “Chinese medicine” and (“dose” or “concentration” or “positive control” or “positive drug”) was used to screen studies with experimental validation. Finally, all screened studies underwent careful evaluation using inclusion and exclusion criteria for case study introduction (Figure 1A, Supplementary Materials Table S1).

A total of 7288 network pharmacology-related records were found in the PubMed database. For studies combining network pharmacology with omics and network pharmacology with artificial intelligence, there were 808 and 773 records in PubMed, respectively, for further introduction (Figure 1A, Supplementary Materials Table S2). Searching with the terms “network pharmacology” and “Chinese medicine” yielded 6773 records. Most lacked experimental validation of network pharmacology predictions or a rigorous design in the pharmacological validation. Critical information such as appropriate controls, full taxonomic validation of investigated material, and dose ranges was omitted in the validation, failing network pharmacological research standards [19]. Thus, we critically assessed these studies using the methods listed in (Supplementary Materials Table S1). Searching with validation-related terms (i.e., “dose”, “concentration”, “positive control”, or “positive drug”) reduced the number of records to 239. To confirm scientifically validated applications of network pharmacology in TCM research, we evaluated these studies and selected the 79 qualified cases for example analysis (Figure 1A, Supplementary Materials Table S2). Word analysis of titles and abstracts revealed high-frequency terms: targets, network, analysis, pharmacology, treatment, pathway, active, and Chinese medicine (Figure 1B). Despite 515 reviews, 92.95% (6773/7288) of articles focused on NP applied to TCM, followed by metabolomics, transcriptomics, lipidomics, proteomics, gut microbiota, and genomics (Figure 1C). NP-related studies increased significantly in recent years. Among these, TCM-related applications (theory, prescription, and herbs) accounted for 40.12% (2924/7288) of publications in 2024, a 28-fold increase from 10 years prior (106/7288) (Figure 1D). This indicates a growing interest in and a proven feasibility of using this method for TCM research.

## 3. Summary of Processes and Resources in Network Pharmacology

**Figure 2:**
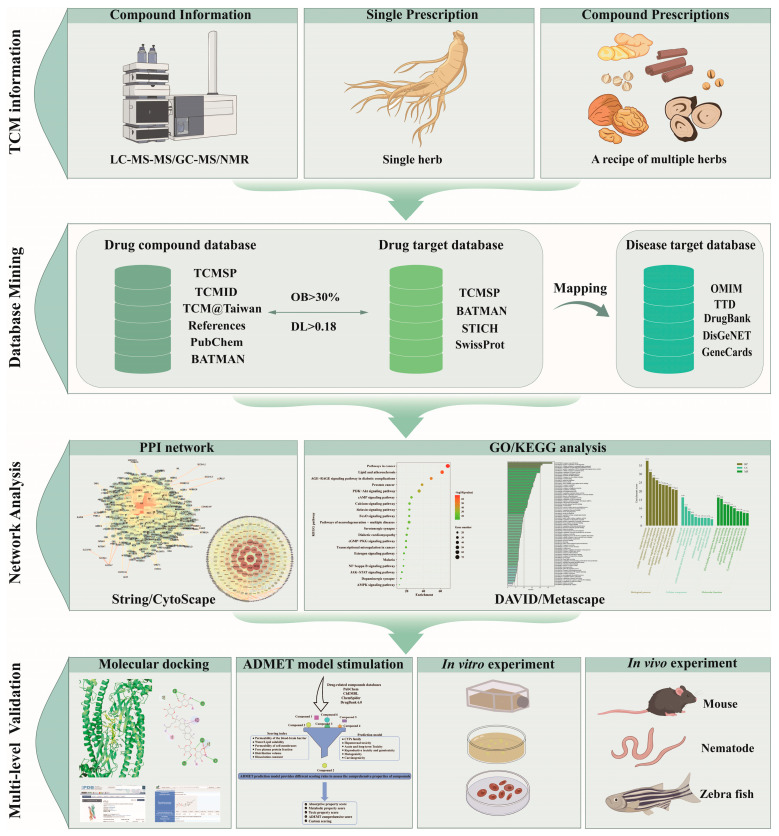
Research pipeline of network pharmacology analysis. TCMSP: Traditional Chinese Medicine Database and Analysis Platform; TCMID: TCM Integrated Database; BATMAN: Bioinformatics Analysis Tool for Molecular Mechanism of TCM; OMIM: Online Mendelian Inheritance in Man database; TTD: Therapeutic Target Database; OB: oral bioavailability; DL: drug-like properties; PPI: protein–protein interaction; STRING: Search Tool for the Retrieval of Interacting Genes/Proteins; GO: Gene Ontology; KEGG: Kyoto Encyclopedia of Genes and Genomes; DAVID: Database for Annotation, Visualization, and Integrated Discovery.

NP employs a systematic approach to elucidate the multi-target mechanisms of TCM. The methodology comprises three integrated stages: (1) constructing networks by collecting TCM compound data through analytical techniques and mining drug/disease targets from databases; (2) analyzing interactions using network topology principles to predict pharmacological effects; and (3) verifying results through molecular docking, absorption, distribution, metabolism, excretion, and toxicity (ADMET) modeling, and in vivo/in vitro experiments (Figure 2).

### 3.1. Core Workflow

In network construction, researchers obtain compound information from TCM prescriptions and integrate drug/disease data from biological databases including the Traditional Chinese Medicine Database and Analysis Platform (TCMSP) [20], PubChem [21], GeneCards [22], and Integrative Pharmacology-based Research Platform of Traditional Chinese Medicine (ETCM) [23]. Additional resources such as OMIM, Therapeutic Target Database (TTD) [24], and KEGG are widely utilized. Data integration leverages known drug–target–disease relationships visualized through software like Cytoscape v3.10.2 [25] or platforms including TCM-Suite [26] and SoFDA [27] (Table 1). For instance, Cytoscape with ClueGo plugin analyzed biological pathways of TCM volatile components against glioma targets [25], while TCMSP-sourced component data combined with Cytoscape visualization revealed Erchen decoction’s mechanism against fatty liver disease via networks of “active components-targets” [28].

### 3.2. Screening Criteria and Network Analysis

During database mining (Figure 2), critical filters were applied: oral bioavailability (OB ≥ 30%) indicates the systemic absorption proportion, where high OB enhances therapeutic efficacy (e.g., N-XBSD nanoparticles increased OB of kukoamine B/mulberroside A), as a higher proportion reaches target sites [53,54]. Additionally, OB provides substantial information about active drug substances. Drugs with appropriate bioavailability enhance absorption while preventing potential toxicity from elevated blood drug concentrations [54]. Drug likeness (DL) quantifies compound similarity to known drugs. Lipinski’s Rule of Five evaluates the DL value of a compound [55]. Compounds with favorable DL values typically exhibit superior pharmacokinetics and meet ADMET requirements, which enhances their drug development potential [56]. Thus, we applied the following screening criteria to Chinese medicine compounds: OB ≥ 30% and DL ≥ 0.18. These thresholds exclude poorly suited molecules while reducing time and costs. These thresholds optimize candidate selection. Subsequent network analysis involves comparative target assessment using the STRING database [47] (parameters: Homo sapiens, confidence > 0.9) to build protein–protein interaction (PPI) networks. Functional enrichment (GO/KEGG) of intersecting targets identified therapeutic pathways, as demonstrated in a study on Sancao Yuyang decoction, where 186 targets revealed HIF-1 signaling hubs (HIF1α, MMP9) [57]. Topological analysis examined node centrality (degree, betweenness) to identify critical targets, exemplified by a study on andrographolide asthma, where IL-6/MMP9 hubs and Th17 pathways were validated [58].

### 3.3. Validation Experiments

A topological analysis of the network reveals the predicted targets, and the results require verification. Common verification methods include molecular docking, ADMET model simulation, and in vivo and in vitro experiments (Figure 2). Molecular docking simulates ligand–target binding, with SystemsDock identifying swertisin/cryptotanshinone as key components in Naozhenning granules [59], Auto Dock Tools revealing coumarin-dithiocarbamate as the α-glucosidase inhibitor backbone [60], and Discovery Studio confirming ginger’s anti-colon cancer action via PI3K-Akt/EGFR pathways [61]. ADMET predictive models serve as critical frameworks for evaluating compound safety and multi-target mechanisms. This approach is particularly significant for deciphering the pharmacological mechanisms of multi-component TCM formulations [62,63]. By integrating human-derived/humanized tissue functional protein systems as surrogate drug targets with advanced in vitro assays and computational simulations, this methodology enables the systematic mapping of drug interaction networks with endogenous physiological and biochemical factors. For example, in the study of canthaxanthin downregulating EGFR in NSCLC, this approach was applied to map multi-target interactions—including EGFR, SRC, and CASP3—with cancer-related signaling pathways (e.g., PI3K-AKT and MAPK) and apoptotic mechanisms, confirming potent and selective anti-tumor activity through integrated computational simulations (molecular docking and dynamics) and in vitro assays [64]. Such multi-dimensional analysis networks facilitate the rational prioritization of candidate compounds with potential bioactivity. NP predicts the underlying mechanism of TCM in disease treatment; ADMET properties can then screen identified candidates for experimental validation [65,66,67]. ADMET data resources can be analyzed through platforms such as ADMETlab [68], Interpretable-ADMET [69], DataWarrior [70], and MetaTox [71] (Table 2). Finally, in vivo (rat/zebrafish) and in vitro (cell models) validations remain essential, exemplified by the Moluodan concentrated pill downregulating TNF-α/PI3K/p-Akt in gastritis [72] and the Buyang huanwu decoction modulating the AKT1/MAPK1/PIK3CA axis against atherosclerosis [73].

## 4. Application of Network Pharmacology in Mining Bioactive Compounds in TCM Prescriptions

### 4.1. Analyzing a Single Prescription

**Figure 3:**
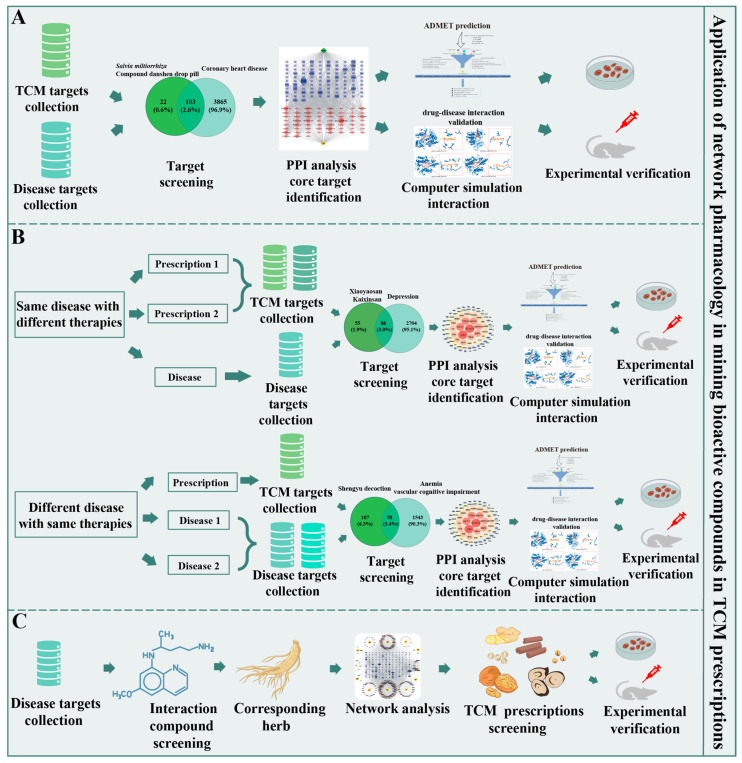
Application of network pharmacology in mining bioactive compounds and elucidating mechanisms of TCM prescriptions. (**A**) Network pharmacology analysis of single prescriptions or compound prescriptions. (**B**) Elucidating the material basis and mechanism of treating “the same disease with different therapies” and “different diseases with the same therapy”. (**C**) Application to reverse pharmacology.

In TCM, each medicinal material has unique therapeutic effects. The complex ingredients of TCM prescription have hindered TCM research. NP identifies drug targets and constructs the drug–target–disease interaction network, which enables the elucidation of single herb/prescription mechanisms (Figure 3A). NP revealed that *Panax notoginseng* saponins (PNSs) exert antidepressant and anxiolytic effects through multi-target mechanisms, including the modulation of neurotransmitter systems (5-HT, GABA, BDNF), suppression of neuroinflammation, inhibition of neuronal apoptosis, and regulation of HPA axis dysfunction, providing a scientific basis for further exploration of PNSs in neurological disorder therapeutics [74]. *Helicobacter pylori* infection has become an international public health problem. The inhibitory activity of *Sanguisorba officinalis* against *H. pylori* was confirmed through NP and in vitro antibacterial activity experiments; 49 potential targets were identified, which concentrated on protein kinase signaling, activity, and binding, as well as pathways in cancer and the TNF signaling pathway [75]. *Saposhnikovia divaricate* has various pharmacological activities, such as inhibiting type I allergy. Through NP analysis, 18 active compounds and 38 intersection targets of *S. divaricate* were obtained; an RBL-2H3 cell degranulation experiment and RT-qPCR analysis showed that *S. divaricate* could inhibit IgE-induced degranulation of mast cells [76].

### 4.2. Analyzing Compound Prescriptions

The multiple components of TCM compound prescriptions can act on several targets and pathways simultaneously. Thus, TCM compound prescriptions hold significant potential for treating comorbidities and chronic diseases such as diabetes, cardiovascular disease, and cancer [77,78,79]. For instance, a study on fenugreek demonstrated this multi-target capacity, where 19 active compounds simultaneously regulated 71 diabetes-related targets—including core genes (ESR1, AKT1, IL6) and pathways (AGE-RAGE, NF-κB)—to improve glucose metabolism via antioxidative, anti-inflammatory, and β-cell protective mechanisms, as validated by integrated docking, dynamics, network analysis, and cell experiments [78]. However, the bioactive ingredients, action mechanisms and targets, and metabolism pathways of TCM compound prescriptions are more complex than those of single prescriptions. Nevertheless, NP can elucidate the active ingredients and action mechanisms of TCM compound prescriptions (Figure 3A). By integrating network medicine analysis with PPI networks and heterogeneous network algorithms, Gao et al. deciphered the molecular linkages between TCM syndromes and hepatocellular carcinoma (HCC), identifying syndrome-specific herbal compounds and validating their therapeutic potential for HCC drug development and precision medicine [80]. By establishing a network medicine framework, Gan et al. elucidated the scientific basis of TCM through the topological linkage between symptom-associated protein modules and herb targets on the human interactome, validated by clinical data, and demonstrated its translational potential for predicting herbal symptom treatments with therapeutic efficacy [81]. Integrated network analysis combined with pharmacological assessment and in vivo validation revealed that Feilike mixture (FLKM) alleviates pneumonia by targeting core anti-inflammatory pathways (TNF/AKT1/IL6/p38MAPK) via its key bioactive components (e.g., resveratrol and stigmasterol), reducing cytokine storm and lung injury, while molecular dynamics confirmed stable ligand–receptor binding (e.g., AKT1–stigmasterol), providing mechanistic evidence for its clinical efficacy [82]. These findings provide scientific support for TCM prescriptions in the clinical treatment of diseases and assist in drug discovery.

### 4.3. Elucidating the Material Basis and Action Mechanisms of Chinese Medicine for Treating the Same Disease with Different Therapies and Different Diseases with the Same Therapy

The “multi-component, multi-target, and multi-pathway” characteristics of TCM have contributed to the development of a unique therapeutic mechanism of “treating the same disease with different therapies and different diseases with the same therapy”. NP analysis aligns with the inherent characteristics of TCM, enabling it to deeply elucidate the materials basis and action mechanisms of Chinese medicine in treating the same disease with different therapies and different diseases with the same therapy (Figure 3B).

The concept of the “treating the same disease with different therapies” was first proposed by “Huangdi Neijing”; it specifically refers to the same disease occurring in different people. Hence, different therapies should be employed for the same disease based on the individual manifestations of the disease. Both Xiaoyaosan powder (XYS) and Kaixinsan powder (KXS) are used to treat depression, exemplifying the principle of “treating the same disease with different therapies”. These formulations exert antidepressant effects by modulating key proteins (including INS, AKT1, TP53, IL6, and CREB1) and regulating critical pathways such as neuroactive ligand–receptor interaction, serotonergic synapse, calcium signaling, cAMP signaling, and cholinergic synapse. Specifically, XYS mediates its antidepressant activity primarily through the PI3K-Akt and MAPK signaling pathways, whereas KXS functions mainly via neuroactive ligand–receptor interaction and serotonergic synapse pathways [83,84]. Despite differing TCM compositions, both formulations target overlapping disease-associated proteins and pathways. Preliminary studies substantiate the pharmacological efficacy of XYS and KXS against depression [85,86]. Collectively, these findings elucidate the molecular mechanisms underlying the “same disease with different therapies” approach from a scientific perspective while also identifying potential candidate compounds for depression treatment.

Anemia is a common hematological disorder, while vascular cognitive impairment (VCI) ranks as the second leading cause of cognitive decline. Notably, both conditions can be treated with the same TCM prescription, exemplifying the concept of “different diseases with the same therapy”. Shengyu decoction (SYD), known for promoting blood circulation, nourishing blood, and soothing nerves, is frequently used to treat anemia and VCI. Integrated NP and LC-MS/MS analysis revealed SYD’s molecular mechanism for anemia: active components (ferulic acid, calycosin, and astragaloside A) act on the PI3K-Akt signaling pathway via AKT1, MAPK1, and MAPK14 [87]. Similarly, NP-guided experimental validation demonstrated that SYD’s active ingredients improve cognitive impairment in VCI model rats by (a) activating the AKT/HIF-1α/VEGF pathway to stimulate cerebrovascular angiogenesis and (b) suppressing p38 MAPK/NF-κB-mediated neuroinflammation [88]. Further illustrating this principle, NP and experimental analysis explored Jiao-Tai-Wan (JTW) in diabetes mellitus with depression. NP predicted JTW’s dual targeting of glucose regulation and depression pathways, subsequently confirmed in vivo: JTW exhibited significant hypoglycemic and antidepressant effects [89]. Collectively, these integrated analyses identified bioactive components with multi-target pharmacological effects, revealing synergistic mechanisms through which a single TCM formula concurrently treats multiple diseases.

Various diseases with similar pathogenic mechanisms can be treated with the same therapeutic agents. Clinical observations have shown the effectiveness of Bushenhuoxue formula (BSHXF) in the treatment of vascular dementia (VD). Researchers employed NP to anticipate the relevant pathways of BSHXF in treating VD and conducted experiments to confirm the association between drug components and the disease; the findings indicated that BSHXF could ameliorate neuronal damage triggered by ischemia in the hippocampus, lower autophagy levels, and thereby enhance learning and memory [90]. Additionally, by combining NP with experimental approaches, researchers found that BSHXF is effective in treating knee osteoarthritis, premature ovarian failure, and chronic kidney disease [91,92,93]. These studies indicate that NP is excellent at elucidating the “multi-component, multi-target, and multi-pathway” characteristics of TCM in treating various diseases, which facilitates the identification of novel compounds capable of synergistically treating multiple diseases and broadens the clinical applications of classic formulas.

### 4.4. Application to Reverse Pharmacology

Reverse pharmacology bridges NP predictions with experimental validation for mining bioactive compounds. According to the reverse pharmacology concept, the targets for a specific disease are first identified, followed by the determination of components interacting with these targets. Subsequently, medicinal materials containing these components are screened through reverse matching and querying, a target–component–drug network is constructed, and TCM prescriptions that alleviate the pathological process are ultimately determined based on the node attributes (Figure 3C). This process is exemplified by diabetes-focused reverse pharmacology studies where key targets (e.g., insulin signaling pathways) were prioritized, leading to the identification of hypoglycemic phytoconstituents—such as allicin in garlic, berberine in barberry, and charantin in bitter gourd—through reverse screening; subsequently, plant sources containing these components were matched, forming a target–component–drug network that validates traditional anti-diabetic prescriptions [94]. The “Jun-Chen-Zuo-Shi” compatibility rules, a fundamental principle governing TCM formula composition that originated as early as “Huangdi Neijing”, must be adhered to in the formation of TCM prescriptions. Notably, NP analysis strategies can be employed to examine the compatibility rules of TCM prescriptions, reveal their pharmacological mechanisms, and provide theoretical support for the research and development of novel TCM preparations [95].

NP-driven reverse pharmacology enables the identification of novel bioactive ingredients, with several typical examples as follows: Xu et al. [96] conducted a screening of targets associated with constipation using DisGeNET and GeneCards databases, identified compounds interacting with these targets through TCMID and TCMSP databases, and subsequently constructed a target–compound–medicine network based on NP; molecular docking was then employed to identify the core prescription capable of alleviating constipation. Gao et al. [97] identified ACE2 and 3CLpro as therapeutic targets for COVID-19 using molecular docking and reverse query methods, discovering six compounds with optimal binding affinity to both targets based on NP. Subsequently, a core herb pair (*Forsythiae fructus* and *Lonicera japonica*) and 16 herbs containing the most active ingredients were selected as candidates for the anti-COVID-19 prescriptions. Lagunin et al. [98] selected genes related to the pathogenesis of vascular dementia (VD) using the PROTEOME, DisGeNET, DISEASES, and DrugBank databases and then combined NP with virtual reverse pharmacology to identify 24 potential drugs that can interact with 10 targets for treating VD, which provided references for the prescription components of anti-VD. Tai et al. [99] identified hub targets and candidate herbs for obesity treatment by integrating bioinformatic analysis and reverse NP and then found ten herbs targeting nine hub targets, among which six herbs were identified as the essential ones. The results are beneficial for constituting promising therapeutic prescriptions to treat obesity.

Currently, reverse pharmacology is still in its early stages of research, and many studies lack experimental verification from a pharmacological perspective. When conducting reverse pharmacology, researchers should prioritize performing essential pharmacological validation experiments, including the integration of multi-omics data such as transcriptomics, proteomics, and metabolomics, along with relevant animal experiments.

## 5. Current Challenges of Applying Network Pharmacology to Drug Screening in TCM

### 5.1. Limitations in Data Resources and Screening Criteria

While network pharmacology (NP) has advanced TCM research through its “multi-components, multi-targets, multi-pathways” approach, critical limitations persist. First, NP remains nascent, its databases rely heavily on single-source research and require dynamic updates to integrate new findings. Second, to maximally exclude ingredients unsuitable for medicinal use and reduce research costs, the selection criteria for the bioactive ingredients based on network databases must be further optimized. The Tanimoto coefficient is used to calculate the drug likeness (DL) of the compounds, with DL thresholds determined by the average DL index of compounds in the Drugbank database [100]. According to TCMSP database recommendations, the current screening criteria for TCM compounds are as follows: oral bioavailability (OB) ≥ 30% and DL ≥ 0.18. However, drugs administered via non-oral routes do not pass through the gastrointestinal tract for absorption but instead enter the bloodstream directly, avoiding first-pass metabolism. Therefore, OB screening becomes irrelevant. Third, predictive results from NP vary significantly due to database discrepancies, increasing risks of false positives/negatives and misaligning between predictions and experimental outcomes.

### 5.2. Reliability Gaps, Clinical Translation Barriers, and Trial Design Challenges

Geographical, harvesting, and processing variations in herbal materials lead to chemical heterogeneity, yet databases often “generalize” TCM compositions without specifying these factors [101]. Core bioactive compounds (e.g., quercetin, kaempferol, and β-sitosterol) recur across prescriptions for diverse diseases, demanding deeper studies to clarify their context-specific efficacy [102,103,104]. These compounds are widely present in different botanical drugs; detailed studies on this phenomenon could help determine the specific efficacy and molecular mechanism of these ubiquitous ingredients, enabling more accurate prediction of the mechanisms of action of different preparations and thereby reducing errors in network analysis. Crucially, NP predictions and preclinical models (in vitro/in vivo) face interspecies metabolic and signaling discrepancies, limiting clinical relevance. The human body is a highly complex system with multidimensional regulatory networks, wherein pharmacodynamic profiles observed in preclinical animal models may exhibit interspecies discrepancies across molecular, cellular, and systemic levels due to fundamental differences in metabolic pathways and receptor signaling cascades. Consequently, pharmacological data measured in vitro and in vivo for a single or compound prescriptions may differ significantly from their effects in humans. To bridge this gap, rigorously designed clinical trials for multi-component TCM drugs must address unique complexities: harmonizing pharmacopeia standards with NP-identified actives, establishing quality control markers reflective of clinical efficacy, and navigating regulatory barriers for botanical drug approval (e.g., FDA-CDER requirements for chemical characterization and batch consistency). Recent publications (e.g., Xuesaitong and Tongxinluo trials in JAMA) exemplify the imperative to align NP-driven research with globally recognized clinical validation frameworks [105,106].

### 5.3. Discrepancies Between Predicted Bioactives and Quality Control

NP-identified disease-specific bioactive compounds often misalign with pharmacopeial quality markers. A notable example is the anticoagulant effects of *Paeoniae* Radix Alba and *Paeoniae* Radix Rubra, which are attributed to compounds like kaempferol and gallic acid, rather than paeoniflorin—the primary quality control marker for both herbs in current pharmacopeial standards [107]. Further studies are required to examine this phenomenon. The concentration of some NP-screening bioactive ingredients predicted to be key in TCM prescriptions may be low in raw herbal materials or finished prescriptions. For instance, NP analysis predicted that three flavonoids—baicalein, wogonin (flavones), and quercetin (flavonol)—in the *Scutellaria baicalensis*-*Sophora japonica* (*Styphnolobium japonicum* (L.) Schott) prescription act on multiple targets and possess significant biological activity; however, their actual concentrations in the final formulation were found to be very low [108]. Addressing this requires integrating NP with advanced analytics (e.g., metabolomics) to redefine quality control paradigms, ensuring markers reflect both chemical consistency and therapeutic bioactivity. Regulatory harmonization is essential to resolve conflicts between traditional markers and NP-guided evidence.

## 6. Synergizing Network Pharmacology, Multi-Omics, and AI to Revolutionize Drug Discovery

The convergence of NP, multi-omics, and AI represents an evolutionary leap in drug discovery, transitioning from reductionist approaches to systems-level interrogation. This paradigm shift is characterized by three transformative capabilities: (i) resolving spatiotemporal heterogeneity via single-cell/spatial omics, (ii) deconvoluting multi-compound synergies through AI-driven network weighting, and (iii) bridging target prediction with functional validation in cellular microenvironments—collectively enabling high-resolution screening of novel bioactive molecules within complex TCM prescriptions.

### 6.1. The Integration of NP with Multi-Omics

With the advent of advanced omics technologies, particularly single-cell sequencing and spatial omics, NP-based mechanistic investigation of TCM has further been implemented and showed a significant potential as the next drug-discovery paradigm.

The combination of NP and transcriptomics provides a new idea for examining the effects of TCM prescriptions on diseases from the perspective of gene expression and transcription regulation. Integrated NP and transcriptome sequencing revealed that luteolin and quercetin exert anti-obesity effects by targeting key genes (*CIDEC*, *Mgll*, *Slc2a4*) and modulating AMPK/AKT signaling pathways, validated through experimental assays, demonstrating their multi-target mechanisms against adipogenic differentiation [109]. Integrated NP, single-cell transcriptomics, and molecular docking was applied to reveal the potential bioactive component (physcion diglucoside) and targets of *Polygonum cuspidatum* Sieb.et Zucc. in treating hepatocellular carcinoma [110]. Integrated network pharmacology, single-cell RNA sequencing, molecular docking, and dynamics simulations reveal that cinobufagin—an active compound from *Venenum bufonis*—inhibits melanoma progression by targeting EGFR, ERBB2, and CDK2 kinases to induce cell cycle arrest [111]. Single-cell transcriptomics enables precise identification of cellular heterogeneity and subtype-specific responses to herbal interventions, while NP, combined with PPI and cell communication networks, systematically maps multi-component, multi-target, and multi-pathway interactions. Their integration overcomes the low-resolution limitations of bulk transcriptomics and static network models, dynamically resolving spatiotemporal regulatory networks in cellular microenvironments, thereby providing a multidimensional framework to decipher the complex interactions between drugs and targets at single-cell resolution [112].

Proteomics can reveal the underlying target proteins and protein biomarkers of organism or cells under normal physiological and disease condition while NP can predict compounds in TCM most likely to interact with proteins targets. Zhang et al. revealed that sanguinarine combats porcine circovirus type 2 (PCV2) by a dual mechanism: upregulating IFIH1/STAT1/MAVS/IRF3/IFITM1 antiviral signaling to enhance viral suppression while inhibiting p38α/JNK pathway activity, thereby reducing PCV2 CAP gene expression and providing a multi-target therapeutic strategy for developing novel anti-PCV2 veterinary drugs based on integrated proteomics and NP [113]. Cai et al. combined NP and proteomics analysis and revealed that ginsenoside Re can prevent myocardial ischemia injury and protect cardiomayocytes from oxidative damage [114]. Xu et al. combined NP, proteomics, Western blot, a mouse xenograft tumor model, and immunofluorescence assays and confirmed the key targets and integrative action mechanisms of *Selaginella doederleinii* against non-small-cell lung cancer [115].

An integrated analysis of NP and metabolome is a potential impetus to discover bioactive compounds, reveal pharmacological mechanisms, and develop novel TCM prescriptions. Licorice flavonoid (LF) exerts gastroprotective effects against ethanol-induced gastric ulcers by modulating 25 metabolic biomarkers linked to amino acid/carbohydrate metabolism and suppressing apoptosis through activation of the PI3K/AKT pathway via key targets (HSP90AA1, AKT1, MAPK1, EGFR, ESR1, PIK3CA), as validated by integrated metabolomics, NP, molecular docking, and experimental assays [116]. A study on the anti-inflammatory mechanism of coumarins in *Peucedanum decursivum* based on spatial metabolomics combined with NP provided a basis for understanding the spatial distribution and anti-inflammatory mechanism of coumarins in *P. decursivum* [117]. The integrated NP and spatial metabolomics approach helped reveal that *Achyrocline satureioides* combats non-small-cell lung cancer (NSCLC) through 6 core components (e.g., quercetin) modulating 32 metabolites and 7 pathways, validated by MALDI-MSI to track tumor-specific metabolic reprogramming and quercetin metabolite accumulation, providing a novel multi-omics framework for herbal anticancer mechanism exploration [118]. The above studies showed that an NP-integrated spatial metabolomics strategy is a promising approach for identifying active components and revealing mechanisms contributing to the pharmacological effects of TCM, which will be beneficial to promote the process of TCM modernization.

Additionally, the integration of NP and lipidomics, as well as of NP and metagenomics (gut microbiota), has also been widely applied to study the action mechanisms of some famous TCM prescriptions against complex diseases such as hyperlipidemia, coronary heart disease, liver injury, and so on [119,120,121,122]. In summary, emerging integrative studies combining NP with multi-omics approaches have identified specific bioactive molecules derived from TCM as promising lead candidates for targeting complex diseases, including chronic atrophic gastritis, chronic kidney disease, and lung cancer [18,123,124,125].

### 6.2. The Integration of NP with AI

Notably, AI has emerged as a pivotal tool for accelerating drug discovery—particularly against complex chronic diseases with high research and development costs—propelling network pharmacology (NP) toward precision TCM research, where AI-driven approaches rapidly identify potential clinical molecules to shorten preclinical phases, thereby elevating NP-based TCM formulation and development to new levels [126,127,128].

The deep integration of AI and NP has significantly overcome core challenges in researching TCM compound formulas. Confronting the difficulty in quantitatively assessing synergistic/antagonistic effects among dozens or even hundreds of components, machine learning (ML) models (e.g., AlphaFold3, artificial neural network, random forest, gradient boosting trees) construct “component–target–disease” weight networks to precisely identify core synergistic components, while deep reinforcement learning optimizes formula compatibility ratios and dosage combinations. This transformative potential stems from ML, particularly deep learning (DL), which provides the essential computational toolkit to overcome NP’s core challenge: mining actionable insights (e.g., synergistic targets, mechanisms, interactions) from massive, heterogeneous data across all NP research stages, from bioactive screening to binding affinity prediction [129]. The revolutionary breakthrough of AlphaFold3 achieves atomic-scale accuracy in predicting 98% of human protein structures (errors < 1 Å), enabling the dynamic simulation of binding energies between TCM components and conformationally dynamic proteins (e.g., tumor mutants), thereby overcoming the limitations of traditional molecular docking [12]. This establishes a closed-loop pipeline: NP screens drugs and targets → AlphaFold3 resolves structures → deep learning performs virtual screening. Concurrently, this integrated AI-NP approach identified five ferroptosis-associated therapeutic targets for osteoporosis (TP53, EGFR, TGFB1, SOX2, MAPK14) and predicted resveratrol as a potential agent binding these targets [130]. It leveraged AlphaFold-predicted structures and molecular docking to elucidate the mechanisms and overcome traditional screening limitations. Complementarily, applying this same pipeline validated the TCM formula Qigui Jiangzhi as augmenting autophagy-facilitated lipid clearance in metabolic-associated fatty liver disease by modulating the AMPK/SIRT1-TFEB axis. NP identifying AMPK/SIRT1 as hubs, and AlphaFold3-based docking revealing compound binding to AMPK [131].

### 6.3. The Integration of NP, Omcis, and AI Is Revolutionizing Drug Discovery

**Figure 4:**
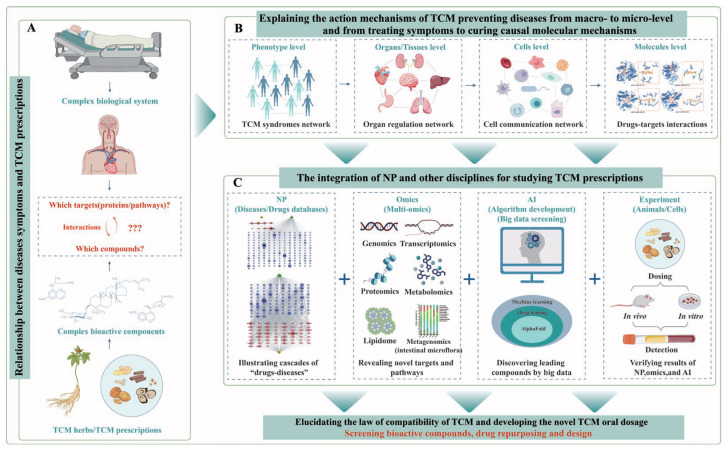
Framework for studying the pharmacological mechanism of TCM preventing disease. (**A**) The hypothesis of the intrinsic relationship between diseases and TCM syndromes. (**B**) The transition of research perspective from the macro-level to micro-level. (**C**) The integration of NP with other disciplines for studying TCM prescriptions and discovering drugs.

The convergence of NP, multi-omics, and AI advances pharmacological studies of TCM prescriptions (Figure 4C) by bridging symptom–prescription relationships (Figure 4A) and decoding molecular mechanisms from macro- to micro-levels (Figure 4B), thereby revolutionizing drug discovery. A machine learning-assisted analysis based on NP and serum metabolomics revealed drug targets and the substance basis of TCM prescription Baiji Wuweizi Granule (BWG) in treating alcoholic liver injury through compound–target–pathway–disease network analysis [132]. Machine learning-based NP can analyze and learn from a large amount of data with different formats (and then summarize underlying patterns), which greatly improves the efficiency of discovering novel drug targets from big data [129]. Similarly, integrating bioinformatics, NP, and AI elucidated the underlying mechanisms (e.g., key targets like ABL1, MAPK1 and pathways like chemokine signaling) by which resveratrol exerts its anti-inflammatory and immunomodulatory effects, highlighting its potential as a therapeutic candidate for rheumatoid arthritis treatment [133].

## 7. Conclusions and Future Perspectives

Despite some limitations, NP has remained crucial in the advancement of TCM and gained increasing attention due to its several advantages, including a systems-level understanding, improved prediction of drug efficacy, discovery of novel drug targets, the development of novel prescriptions, and drug repurposing and design.

An integrative framework has been established to elucidate the mechanisms of disease prevention in TCM and facilitate the discovery of bioactive compounds and drug repurposing and design (Figure 4). This paradigm shift originated from the hypothesis that intrinsic connections exist between disease symptoms and TCM prescriptions, with research perspectives evolving through three critical transitions: (i) from syndrome-based macroscopic phenotyping to molecular-level mechanistic exploration; (ii) from symptom management to causal mechanism targeting; and (iii) ultimately implementing convergent methodologies incorporating NP, multi-omics profiling, and AI to accurately identify bioactive components with defined therapeutic efficacy in TCM formulations.

The integrated analysis has five significant advantages, as follows: (i) comprehensively illustrating the complex biological cascade of “drug–gene–target–disease” networks from the perspective of “multi-components, multi-targets, and multi-pathways”; (ii) revealing novel action targets and signaling pathways of drugs preventing diseases; (iii) discovering reliable lead monomers from Chinese medicines by filtering big data; (iv) providing a scientific basis for the laws of compatibility of TCM and the development of novel oral dosage forms of TCM prescriptions; and (v) facilitating secondary development of classic famous TCM prescriptions and the repurposing of bioactive monomers derived from Chinese medicines. So far, several excellent studies on integrating NP, multi-omics, and AI to investigate monomer botanical drugs and TCM prescriptions have been reported [133,134,135]. In summary, research integrating NP and other disciplines, especially AI and multi-omics, will become a popular trend in TCM studies and further facilitate the clinical translation application of bioactive monomers and TCM prescriptions in the future, which will enhance the global acceptance of TCM products and accelerate novel drug discovery.

## Figures and Tables

**Table 1:** Common databases and software used in network pharmacology research.

Type	Name	Description	Website (Access Date)	Release	Refs.
TCM-related databases	TCMSP	Chinese herbal medicine action mechanism analysis platform and database, including 499 kinds of herbal medicines, providing herbal ingredients and key pharmacokinetic properties, and obtaining the relationship between Chinese herbal medicines, targets, and diseases.	https://tcmsp-e.com/tcmsp.php (1 June 2025)	Monthly	[20]
	ETCM 2.0	This database includes comprehensive information on TCM formulas and their ingredients and provides predictive targets for TCM formulas and their ingredients. It can systematically establish a network of relationships among ingredients, herbs, formulas, targets, and diseases.	http://www.tcmip.cn/ETCM/ (1 June 2025)	2023	[23]
	TCMID 2.0	A comprehensive database with the goal of the modernization and standardization of TCM, including 46,929 prescriptions, 8159 herbal medicines, 43,413 total ingredients, 8182 drugs, and 4633 diseases.	https://bidd.group/TCMID/about.html (1 June 2025)	2017	[29]
	TCMBanK	The largest TCM database. It provides deep learning-based Chinese–Western medicine exclusion prediction.	http://tcm.cmu.edu.tw (1 June 2025, not accessible)	2023	[30]
	HERB	A reference guide database for high-throughput experiments of traditional Chinese medicine, including 12,933 targets, 28,212 diseases, 7263 herbs, and 49,258 ingredients.	http://herb.ac.cn (1 June 2025)	2020	[31]
	HIT 2.0	Comprehensive Chinese herbal medicine ingredient index database; molecular target information, including proteins that are directly/indirectly activated/inhibited; protein conjugates and substrates; products which are the enzymes of these compounds.	http://hit2.badd-cao.net/ (1 June 2025)	2021	[32]
	ITCM	The largest-to-date online TCM active ingredient-based pharmacotranscriptomic platform on integrated TCM for effective screening of bioactive compounds.	http://itcm.biotcm.net (1 June 2025)	2022	[33]
	CPMCP	A TCM-related database collecting components, indications, and contraindications originating from TCM.	http://cpmcp.top/ (2 Nov 2022, not accessible)	2022	[34]
	SymMap	An integrative database of TCM enhanced by symptom mapping.	http://www.symmap.org/ (1 June 2025)	2019	[35]
Drug-related databases	PubChem	Public chemical information resources to analyze the biological activity of small chemical molecules.	https://pubchem.ncbi.nlm.nih.gov (1 June 2025)	2021	[21]
	ChEMBL	Open large-scale biological activity database, including target annotation and drug metabolism pathways.	https://www.ebi.ac.uk/chembl (1 June 2025)	2019	[36]
	ChemSpider	Free chemical structure database, providing fast text and structure search of 67 million structures.	http://www.chemspider.com (1 June 2025)	2024	[37]
	DrugBank 6.0	This database provides detailed drug data and drug target information, as well as comprehensive molecular information about their mechanisms.	https://go.drugbank.com (1 June 2025)	2023	[38]
Protein-related databases	UniProt	The protein database with the most information and resources, providing protein sequences with functional information annotations.	https://www.uniprot.org (1 June 2025)	2023	[39]
	MINT	Public repository of protein interactions.	https://mint.bio.uniroma2.it (1 June 2025)	2012	[40]
	HPO	This database provides phenotypic information about human diseases, containing more than 13,000 terms and more than 156,000 notes on genetic diseases.	https://hpo.jax.org (1 June 2025)	2021	[41]
Disease-related databases	GeneCards	This database provides comprehensive annotated and predicted human genes, including genome, transcriptome, proteome, and related functional information.	https://www.genecards.org (1 June 2025)	2024	[22]
	CTD	This database provides information on chemical–gene/protein interactions, chemical–disease and gene–disease relationships and helps elucidate potential mechanisms of environmental impact on diseases.	https://ctdbase.org (1 June 2025)	2023	[42]
	TTD	This database provides information about the main targets of drugs.	http://db.idrblab.net/ttd/ (1 June 2025)	2022	[43]
	DisGeNET	This database includes 1,134,942 gene–disease associations (GDAs) and 369,554 variant–disease associations (VDAs).	https://www.disgenet.org (1 June 2025)	2020	[44]
	MalaCards	An integrated database of human maladies and their annotations.	https://www.malacards.org (1 June 2025)	2023	[45]
	CHD@ZJU	This database provides a network-based study platform on coronary heart disease.	http://tcm.zju.edu.cn/chd/ (not accessible)	2023	[46]
Protein interaction database	STRING v12	Protein networks with directionality of regulation.	https://string-db.org (1 June 2025)	2024	[47]
	BioGRID	Archive of genetic and protein interaction data from model organisms and humans.	https://thebiogrid.org/ (1 June 2025)	2021	[48]
	IntAct	An open-source database and analysistool for molecular interaction data.	https://www.ebi.ac.uk/intact/home (1 June 2025)	2022	[49]
Software or platform	Cytoscape	This database integrates bio-molecular interaction networks with high-throughput expression data and other molecular states into a unified conceptual framework.	https://cytoscape.org (1 June 2025)	2024	[25]
	TCM-Suite	A comprehensive and holistic platform for traditional Chinese medicine component identification and network pharmacology analysis.	http://TCM-Suite.AImicrobiome.cn (1 June 2025)	2022	[26]
	SoFDA	Ontology characterization, enrichment analysis, and similarity calculation-based evaluation of disease–syndrome–formula associations.	http://www.tcmip.cn/Syndrome/front/ (1 June 2025)	2023	[27]
	Metascape	This database is able to perform GO/KEGG enrichment analysis of genes, including a large number of databases and tools for gene annotation and gene enrichment analysis.	https://metascape.org/ (1 June 2025)	2019	[50]
	BATMAN- TCM 2.0	The first online bioinformatics analysis platform specially designed for studying the molecular mechanisms of TCM.	http://bionet.ncpsb.org.cn/batman-tcm/index.php (1 June 2025)	2024	[51]
	LTM-TCM	A standardized platform for studying TCM mechanisms, providing reverse docking and ADME prediction analyses.	http://cloud.tasly.com/#/tcm/home (not accessible)	2022	[52]

**Table 2:** Data sources and system analysis of ADMET.

Name	Description	Website (Access Date)	Release	Refs.
ADMETlab 2.0	An integrated online platform for accurate and comprehensive predictions of ADMET properties.	https://admetmesh.scbdd.com/ (1 June 2025)	2021	[68]
Interpretable-ADMET	A web service for ADMET prediction and optimization based on deep neural representation.	http://cadd.pharmacy.nankai.edu.cn/interpretableadmet/ (1 June 2025)	2022	[69]
DataWarrior	Explores the compound space in an interactive way by visualizing the spatial structure of compounds or pharmacophores based on vector or non-vector descriptors.	http://www.openmolecules.org/datawarrior/download.html (1 June 2025)	2019	[70]
MetaTox 2.0	Predicts the toxicity of metabolites in the body based on the structure of the compound.	https://www.way2drug.com/metatox (1 June 2025)	2023	[71]

## Data Availability

Not applicable.

## Supplementary Materials

The following supporting information can be downloaded at https://www.mdpi.com/article/10.3390/ph18071074/s1, Table S1: Inclusion and exclusion criteria used for the data analysis on network analysis applied in TCM; Table S2: Literature collection and statistics result (provided in Excel format).

## Abbreviations

TCM: traditional Chinese medicine; AI: artificial intelligence; NP: network pharmacology; GC-MS: gas chromatography–mass spectrometry; LC-MS/MS: liquid chromatography–tandem mass spectrometry; TCMSP: Traditional Chinese Medicine Systems Pharmacology Database and Analysis Platform; PI3K/AKT: phosphatidylinositol 3-kinases/protein kinase B; GNN: Graph Neural Networks; Q-markers: quality markers; ADMET: absorption, distribution, metabolism, excretion, and toxicity; NMR: nuclear magnetic resonance; ETCM: the Encyclopedia of Traditional Chinese Medicine; ECD: erchen decoction; OMIM: Online Mendelian Inheritance in Man; TTD: Therapeutic Target Database; KEGG: Kyoto Encyclopedia of Genes and Genomes; TCMID: TCM Integrated Database; BATMAN: Bioinformatics Analysis Tool for Molecular Mechanism of TCM; OB: oral bioavailability; DL: drug-like properties; PPI: protein–protein interaction; STRING: Search Tool for the Retrieval of Interacting Genes/Proteins; GO: Gene Ontology; DAVID: Database for Annotation, Visualization, and Integrated Discovery; HERB: A high-throughput experiment- and reference-guided database of traditional Chinese medicine; HIT: Herbal Ingredients’ Targets Platform; ITCM: Integrated Traditional Chinese Medicine; CPMCP: Chinese patent medicine and compound prescription; MINT: a Molecular INTeraction database; HPO: human phenotype ontology; CTD: Comparative Toxicogenomics Database; N-XBSD: nanoparticles in Xie-Bai-San decoction; AUC: area under the curve; SCYYD: Sancao Yuyang decoction; HIF-1: hypoxia-inducible factor 1; HIF1α: hypoxia-inducible factor 1 alpha; MMP9: matrix metalloproteinase 9; AG: andrographolide; IL-6: interleukin-6; Th17: intestinal IL-17-producing T helper; EGFR: epidermal growth factor receptor; NSCLC: non-small-cell lung cancer; SRC: sparse representation-based classifier; CASP3: caspase-3; MAPK: mitogen-activated protein kinases; MLD: Moluodan concentrated pill; TNF-α: tumor necrosis factor alpha; BYHWT: Buyang huanwu decoction; PIK3CA: p110alpha subunit of PI3K; PNS: Panax notoginseng saponins; 5-HT: 5-hydroxytryptamine; GABA: gamma-aminobutyric acid; BDNF: brain-derived neurotrophic factor; HPA: hypothalamic–pituitary–adrenal; RBL-2H3: rat basophil leukemia cells; RT-qPCR: reverse transcription–quantitative polymerase chain reaction; IgE: anti-immunoglobulin E; ESR1: estrogen receptor 1; AGE-RAGE: advanced glycation end products-receptor of advanced glycation end products; NF-κB: nuclear factor-kappa B; HCC: hepatocellular carcinoma; FLKM: Feilike mixture; XYS: Xiaoyaosan powder; KXS: Kaixinsan powder; INS:/inertial navigation system; TP53: tumor protein 53; CREB1: cAMP responsive element binding protein 1; cAMP: cyclic adenosine monophosphate; VCI: vascular cognitive impairment; SYD: Shengyu decoction; JTW: Jiao-Tai-Wan; BSHXF: Bushenhuoxue formula; VD: vascular dementia; ACE2: Angiotensin-converting enzyme 2; 3CLpro: 3C-like protease; COVID-19: coronavirus disease 2019; FDA-CDER: Center for Drug Evaluation and Research (CDER), U.S. Food and Drug Administration; JAMA: *The Journal of the American Medical Association*; CIDEC: cell death-inducing DFF45-like effector C; RNA: ribonucleic acid; DPP4: dipeptidyl peptidase-4; CD40LG: CD40 ligand; BCL2: B cell lymphoma 2; PCV2: porcine circovirus type 2; FIH1: factor inhibiting hypoxia-inducible factor-1; STAT1: signal transducer and activator of transcription 1; MAVS: mitochondrial antiviral signaling protein; IRF3: interferon regulatory factor 3; IFITM1: interferon-induced transmembrane protein 1; CAP: capsid protein; LF: licorice flavonoid; HSP90AA1: heat shock protein 90 alpha family class A member 1; MALDI-MSI: matrix-assisted laser desorption/ionization mass spectrometry imaging; ML: machine learning; DL: deep learning; TGFB1: transforming growth factor beta 1; SOX2: Sex determining region Y-box 2; AMPK: AMP-activated protein kinase; SIRT1: silent information regulator sirtuin 1; TFEB: transcription factor EB; BWG: Baiji Wuweizi Granule; ABL1: v-abl Abelson murine leukaemia viral oncogene homologue 1.
