# Supplementary material for: Network Pharmacology-Driven Sustainability: AI and Multi-Omics Synergy for Drug Discovery in Traditional Chinese Medicine
Source: Pharmaceuticals (Basel). 2025 Jul 21;18(7):1074. doi: 10.3390/ph18071074 (PMC12298991; doi:10.3390/ph18071074)
Supplement: Supplementary file 1 [file pharmaceuticals-18-01074-s001.zip › Supplementary Table 1.pdf]

Table S1 Inclusion and exclusion criteria used for the data analysis on network analysis applied in TCM.

Number	Inclusion criteria	Exclusion criteria
1	English	Languages other than English
2	Studies conducted in combination with experimental pharmacology (*in vitro* or *in vivo*)	Studies conducted without the validation of experimental pharmacology (*in vitro* or *in vivo*)
3	Pharmacological validation involving appropriate controls, full taxonomic validity of the material under investigation, models relevant to the question being researched, dose range tested, and other basic pharmacological data	Pharmacological validation without appropriate controls, full taxonomic validity of the material under investigation, models relevant to the question being researched, dose range tested, or other basic pharmacological data
4	Compounds, herbal (medical) product, or preparations of pharmacological relevance	Compounds, herbal (medical) product, or preparations lacking pharmacological relevance
5	Activities being able to be assessed from a pharmacological perspective	Activities unable to be assessed from a pharmacological perspective
6	Anti-oxidant activity evaluated by pharmacological experiments other than chemical assays such as FRAP, ABTS, DPPH, and Trolox equivalent antioxidant capacity assays.	Antioxidant activity evaluated by only chemical assays such as FRAP, ABTS, DPPH, and Trolox equivalent antioxidant capacity assays.
7	Anti-microbial activity evaluated by *in vitro* or *in vivo* experiments other than single disc diffusion experiments.	Antimicrobial activity evaluated by only disc diffusion experiments.
8	Anti-inflammation activity evaluated by a larger pharmacological-phytochemical study other than single rat hind paw oedema model	Anti-inflammation activity evaluated by only rat hind paw oedema model
